# Household tuberculosis contact investigation in a tuberculosis-prevalent country: Are the tuberculin skin test and interferon-gamma release assay enough in elderly contacts?

**DOI:** 10.1097/MD.0000000000009681

**Published:** 2018-01-19

**Authors:** Jung Seop Eom, Insu Kim, Won-Young Kim, Eun-Jung Jo, Jeongha Mok, Mi-Hyun Kim, Kwangha Lee, Ki Uk Kim, Hye-Kyung Park, Min Ki Lee

**Affiliations:** a Department of Internal Medicine, Pusan National University School of Medicine; b Biomedical Research Institute, Pusan National University Hospital, Busan, Korea.

**Keywords:** elderly, interferon-gamma release test, latent tuberculosis, tuberculin test, tuberculosis

## Abstract

The high background rates of positive results on the tuberculin skin test (TST) and interferon-gamma release assay (IGRA) sometimes confuse the investigation of tuberculosis (TB) contact in TB-prevalent countries, particularly in elderly contacts. The aim was to investigate the predictive value of TST and IGRA for diagnosing latent TB infection (LTBI) in elderly household contacts in South Korea.

In this retrospective study, TST and IGRA results of household contacts of suspected pulmonary TB patients were reviewed according to the index patient's final diagnosis (TB group: culture-confirmed pulmonary TB, non-TB group: pulmonary disease other than TB).

A total of 249 contacts were included in the analysis (188 in the TB group and 61 in the non-TB group). In the TB group, TST and IGRA were positive in 42.6% and 45.7% of contacts, respectively. In the non-TB group, TST and IGRA were positive in 32.8% and 23.0% of contacts, respectively. TST did not show any differences between the TB and non-TB groups for any age group, whereas IGRA showed differences between the 2 groups for those ages 18 to 39 and 40 to 59 years. However, there were no significant differences between the groups for the ≥60 years old group.

In elderly contacts, neither TST nor IGRA showed clear discrimination of positivity between the groups. Further studies are needed to predict which elderly contacts are at risk for progression to active TB as well as to accurately detect recent *Mycobacterium tuberculosis* infection in this vulnerable population.

## 1 Introduction

Early detection and appropriate treatment of active tuberculosis (TB) patients are the foundation of national TB control programs worldwide. However, identifying and treating latent TB infection (LTBI) in those at risk for progression to active disease are essential to reduce the incidence rate and eliminate TB, particularly in high-resource settings.^[1,2]^ Close contact with TB patients is a well-recognized risk factor for *Mycobacterium tuberculosis* infection. Specifically, household contacts are at the greatest risk for LTBI because they share the same airspace with TB patients in a congregated setting for a prolonged period of time.^[3]^ Furthermore, the likelihood of progression from LTBI to TB in household contacts is usually higher than in the general population.^[4]^ Therefore, household contacts of TB patients are considered a high-priority population for contact investigation.^[3]^

Traditionally, the tuberculin skin test (TST) has been used to diagnose LTBI. However, this method has several limitations, including a tendency to give false-positive results in Bacillus Calmette–Guérin (BCG)-vaccinated persons. Over the past decade, the interferon-gamma release assays (IGRA) has been introduced into clinical practice for the diagnosis of LTBI. In this test, interferon-gamma is measured in vitro in response to TB-specific antigen stimuli. IGRA shows better specificity in the BCG-vaccinated population because unlike TST it is unaffected by BCG vaccination.^[5,6]^

However, the predictive value of TST, and even of IGRA, is sometimes questionable because the prevalence of LTBI based on these tests increases with age in TB-prevalent countries.^[7–10]^ This high background rate of positive results raises concerns about the ability to accurately distinguish between recent and previous *M tuberculosis* infection, particularly in elderly contacts, which thus confuses decisions regarding LTBI treatment. However, sufficient data regarding the utility of TST and IGRA for elderly contacts are still lacking in countries where TB is prevalent and BCG vaccination is mandatory.

The present study investigated the predictive value of TST and IGRA for diagnosing LTBI among elderly household contacts in South Korea (the estimated TB incidence was 80 per 100,000 population in 2015) by comparing results for pulmonary TB and non-TB pulmonary disease contacts. We also evaluated factors that influence positive results for TST and IGRA in household contacts of pulmonary TB.

## 2 Methods

### 2.1 Study design and subjects

This retrospective study was conducted at Pusan National University Hospital, a university-affiliated tertiary care hospital in Busan, South Korea, that has 1400 beds. Household contacts (≥18 years old) of suspected pulmonary TB patients who underwent contact investigations were screened between January 2013 and December 2014. Of these, contacts with both TST and IGRA results were included. Contacts with a previous medical history of TB or LTBI or with an indeterminate IGRA result were excluded. Contacts of TB patients with negative culture results were also excluded because their index patients might not have been contagious. These index patients underwent TB treatment because they exhibited clinical and radiological findings compatible with TB that improved after treatment, and were thus finally diagnosed with TB. A household contact was defined as an individual who had resided in the same house with an index patient for at least 3 months prior to the diagnosis of TB in the index patient.

The contacts were divided into 2 groups according to the index patient's final diagnosis: a TB group (the index patient's diagnosis was culture-confirmed pulmonary TB) and a non-TB group (the index patient was initially suspected of having pulmonary TB, but the final diagnosis was a pulmonary disease other than TB, e.g., bronchiolitis, bronchitis, or pneumonia, that improved with antibiotics that were not effective against *M tuberculosis*).

This study was conducted with approval from the Institutional Review Board of Pusan National University Hospital (1701-038-001), but informed consent was not obtained from each contact because of the retrospective nature of the study. Our study protocol was part of a routine practice of contact investigation in our hospital and had no effect on the diagnosis or treatment of LTBI in contacts.

### 2.2 Data collection

The following data were collected from each contact's medical records: age, sex, comorbidities, history of BCG vaccination, proximity to the index patient (living in the same room or a different room), and TST and IGRA results. Immunocompromised status was defined as human immunodeficiency virus infection; silicosis; diabetes mellitus; chronic kidney disease requiring renal replacement therapy; malignant disease; or receipt immunosuppressive agents, tumor necrosis factor-α antagonists, or systemic corticosteroid treatment.

We also investigated data from index patients’ medical records: age, sex, final diagnosis, cough on day of 1st hospital visit, cavitation on chest radiography or computed tomography, sputum acid-fast bacilli (AFB) staining, and culture result.

### 2.3 TST and IGRA test procedures

Each TST was performed according to the Mantoux method. Well-trained nurses injected 0.1 mL (2 tuberculin units) tuberculin-purified protein derivative RT 23 (Statens Serum Institute, Copenhagen, Denmark) into the forearm of the contact.^[11,12]^ The transverse diameter (mm) of the induration was measured 48 to 72 hours after injection. TST reaction was defined as positive if the induration diameter was ≥10 mm, according to Korean guidelines for TB, regardless of BCG vaccination status.^[13]^

IGRA was performed using the T-spot.*TB* test (Oxford Immunotec Ltd, Abingdon, UK) according to the manufacturer's instructions.^[14,15]^ Immediately before TST, 6 mL peripheral venous blood was drawn from each contact. Processed peripheral blood mononuclear cells were incubated in each well with medium alone (as the nil control), phytohemagglutinin (as the positive control), and early secreted antigenic target-6/culture filtrate protein-10 for 20 hours. The number of spots was counted using an automated AID ELISPOT plate reader (AID Systems, Strasbourg, Germany). Tests were scored as positive if early secreted antigenic target-6- or culture filtrate protein-10-stimulated wells contained at least 6 spots more than the nil control well and if this number was at least twice that of the nil control well. Tests were considered indeterminate if the positive control well contained <20 spots or if the negative control well contained >10 spots.

### 2.4 Statistical analysis

Data are presented as means ± standard deviations for continuous variables and as frequencies (with percentages) for categorical variables. Continuous variables were compared using independent-samples *t* tests, and categorical variables were compared using Pearson chi-square test or Fisher exact test. The kappa statistic (κ) was used to evaluate the concordance between TST and IGRA. To evaluate the trends in TST and IGRA results by age, we used the chi-square test. Logistic regression analysis was performed for predictors associated with positive TST and IGRA results in subjects. All data were analyzed using SPSS version 17.0 (SPSS, Chicago, IL). In all analyses, *P* < .05 indicated statistical significance.

## 3 Results

### 3.1 Baseline characteristics of the participants

**Figure 1:**
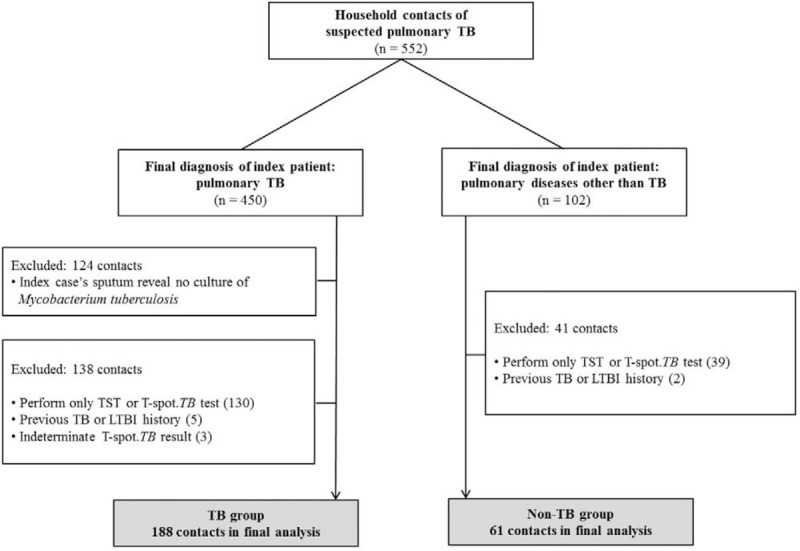
Flowchart of study participants. LTBI = latent tuberculosis infection, TB = tuberculosis, TST = tuberculin skin test.

A total of 552 contacts of 317 index patients with suspected pulmonary TB were screened for inclusion during the study period. After application of the criteria outlined above, 249 contacts (188 in the TB group and 61 in the non-TB group) were included in the final analysis (Fig. 1). The most common diagnoses of index patients (n = 43) in the non-TB group were bronchiolitis and bronchitis (n = 25), followed by nontuberculous mycobacterial lung disease (n = 11), pneumonia (n = 3), lung cancer (n = 3), and benign lung nodule (n = 1). One contact in the TB group had active pulmonary TB during the contact investigation.

Table 1 shows the baseline characteristics of the contacts and index patients in the 2 groups. The mean age of the contacts was 48.8 ± 17.2 years, and 34.1% were male. A total of 89.6% of the contacts had received BCG vaccination. The rate of positive AFB smear of the index patient's sputum was 59.8% in the TB group. There were no significant differences in characteristics of contacts or index patients between the groups.

**Table 1:**
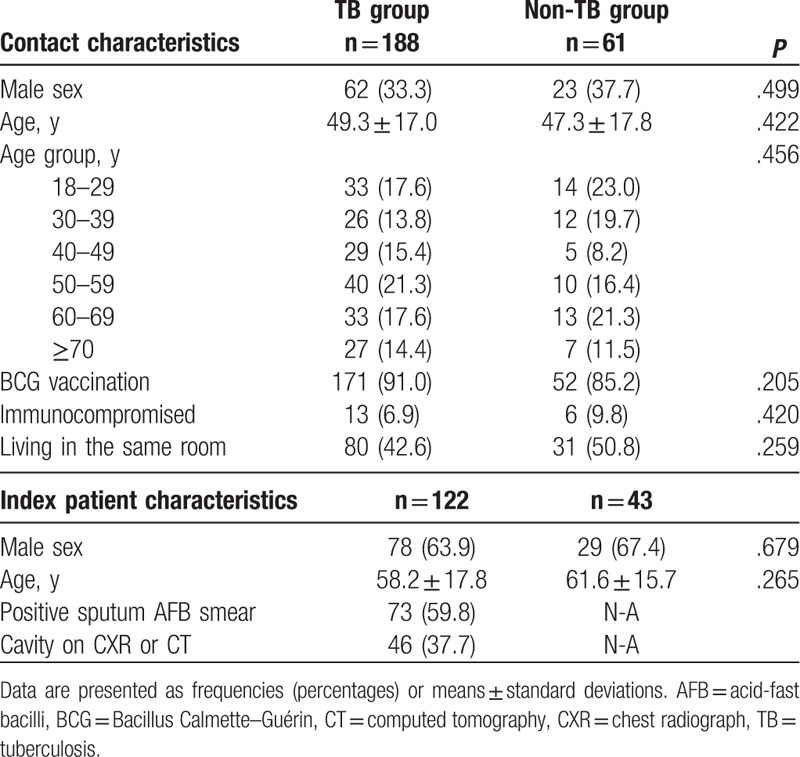
Baseline characteristics of contacts and index patients.

### 3.2 Comparison of TST and IGRA results between the groups

TST and IGRA were performed 13.0 ± 20.8 days after the index patient was suspected of having pulmonary TB. In the TB group, TST and IGRA were positive in 42.6% and 45.7% of contacts, respectively. In the non-TB group, TST and IGRA were positive in 32.8% and 23.0% of contacts, respectively (Table 2). Both TST and IGRA results showed a higher tendency in the TB group than the non-TB group, but only the IGRA result was significantly different between the groups (*P* = .002; Table 2). The overall agreement between TST and IGRA in the TB group and non-TB group was 72.3% and 83.6%, respectively (Table 3). Kappa coefficients showed moderate agreement in both groups (κ = .440 in the TB group, κ = .597 in the non-TB group).

**Table 2:**
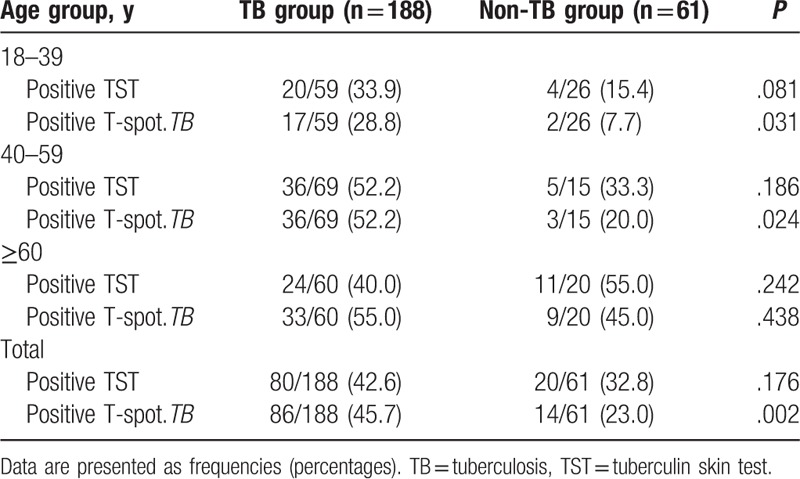
Tuberculin skin test and T-spot.*TB* test results by age group.

**Table 3:**
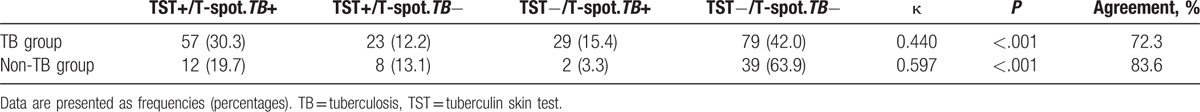
Concordance between tuberculin skin test and T-spot.*TB* test by group.

### 3.3 TST and IGRA results by age group

**Figure 2:**
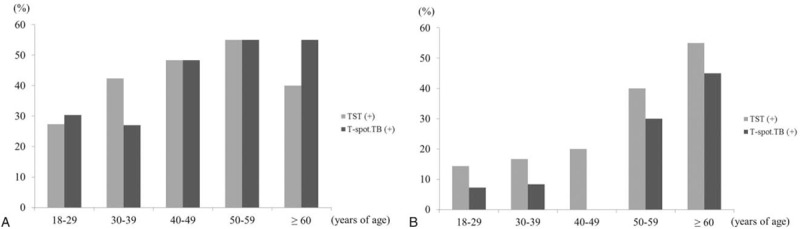
Tuberculin skin test and T-spot.*TB* test positivity by age. (A) TB group. (B) Non-TB group. TB = tuberculosis, TST = tuberculin skin test.

In the non-TB group, both TST and IGRA positivity increased significantly with age (*P* = .005 for TST, *P* = .003 for IGRA; Fig. 2). In the TB group, positivity on both tests showed an increasing trend with age, but this trend did not reach statistical significance for TST (*P* = .271 for TST, *P* = .003 for IGRA; Fig. 2). TST results did not show any differences between the TB and non-TB groups according to age, whereas IGRA showed differences between the 2 groups in contacts 18 to 39 and 40 to 59 years old. However, there were no significant differences between the 2 groups in those ≥60 years old (Table 2).

### 3.4 Predictors of positive TST and IGRA results in the TB group

Logistic regression analysis was performed to identify predictors associated with positive TST and IGRA results in the TB group. In multivariate analyses, male sex and AFB smear positivity of the index patient were associated with a positive TST result (Table 4), whereas proximity (living in the same room) and AFB smear positivity of the index patient were independent risk factors for a positive IGRA result (Table 5).

**Table 4:**
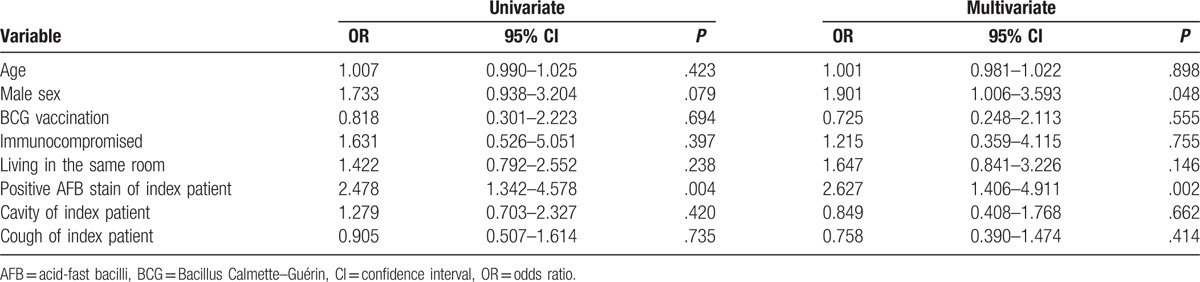
Predictors associated with a positive tuberculin skin test in contacts of TB group.

**Table 5:**
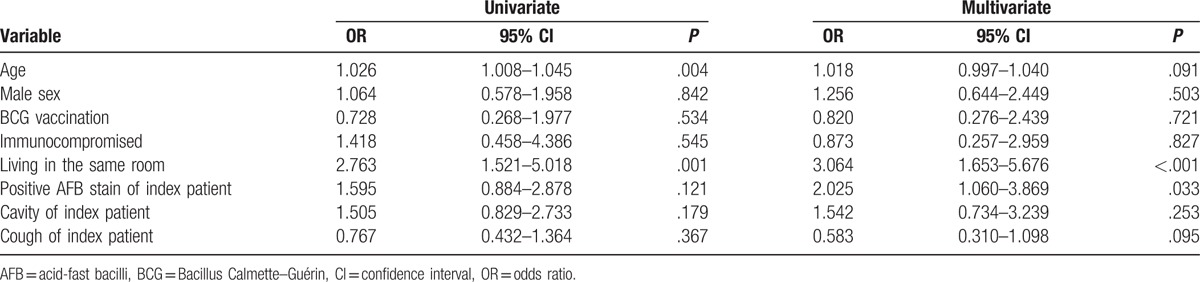
Predictors associated with a positive T-spot.*TB* test in contacts of TB group.

## 4 Discussion

In the present study, the IGRA positivity rate was clearly higher in contacts of pulmonary TB patients than in contacts of those with pulmonary diseases other than TB. Conversely, although there was a greater trend toward TST positivity in contacts of TB patients, there were no significant differences between the 2 groups. This result is consistent with previous studies of QuantiFERON-TB Gold-in-tube for contacts and noncontacts of TB patients in South Korea^[7,16]^ and suggests that, like the QuantiFERON-TB Gold-in-tube, the T-spot.*TB* test may be more reliable than TST for diagnosing LTBI in household contacts of TB patients in countries, where BCG vaccination is mandatory. Unlike in previous studies, both TST and IGRA results were available for same contacts in our study, and this strengthened the evidence for the utility of IGRA.

However, in elderly contacts (≥60 years old), there were no significant differences in positivity between the TB and non-TB groups, even with IGRA. This may be due to the high background positive rates of IGRA in the elderly population in South Korea.^[7]^ Our results highlight a dilemma in making treatment decisions for LTBI in elderly contacts because elderly contacts have vulnerable immunity and therefore develop TB more easily than younger contacts when they contract LTBI, and they may also have frequent adverse drug reactions during LTBI treatment.

Given that LTBI affects nearly one-third of all people worldwide,^[17]^ it is important to identify treatment candidates. Recent *M tuberculosis* infection is well-known to be a risk factor for TB development and, therefore, contacts who were infected recently should be given high priority in terms of LTBI treatment.^[3]^ However, the high background test positivity renders it difficult to accurately distinguish between recent and previous *M tuberculosis* infections, especially in elderly contacts living in TB-prevalent countries. Therefore, biomarkers, or novel tests, distinguishing recent from previous infections, or predicting progression from LTBI to active TB, are required. Several studies have shown that the “region of difference 1”-specific immune response of IGRA, immune sensitization to purified protein derivative (as revealed by the TST), microribonucleic acid expression signature, level of D-related human leukocyte antigen-expressing CD4 T cells, and blood monocyte/lymphocyte ratio may be candidate biomarkers.^[18]^ However, the predictive utility of these biomarkers remains unclear; further validation is needed. Also, only limited data on elderly populations are available.

From a clinical perspective, it is important to identify clinical characteristics associated with TB progression in contacts with LTBI. Apart from recent infection and aging per se, immunocompromised status (human immunodeficiency virus infection, diabetes mellitus, chronic kidney disease, or malignant disease) and/or the use of immunosuppressive agents/systemic steroids are well-known risk factors for active TB development.^[19]^ However, the evidence that these risk factors are uniformly in play in elderly contacts is limited. Until these issues have been thoroughly explored via large-scale studies with elderly populations, to avoid confusion clinical parameters or contact situations suggestive of recent infection should be the prime considerations when evaluating elderly contacts. Relevant factors include the infectivity of the index patient, the proximity of the contact to the index patient, and the contact duration and intensity.

In our study, living in the same room as the index patient and AFB smear positivity of the index patient were associated with IGRA positivity. Previous studies found that several factors are associated with IGRA positivity in close contacts of TB patients.^[20–22]^ Advanced age, AFB smear positivity in the index patient, cough of the index patient, longer contact duration, and sharing the same airspace with the index patient are well-known risk factors for LTBI. However, there are insufficient data showing that these risk factors are associated with the progression of LTBI to active TB. As the purpose of contact investigation is to treat high-risk contacts for the development of TB, further evaluations are necessary to determine whether such risk factors for LTBI also contribute to TB development.

This study has several limitations. First, the size of the study population, particularly contacts of non-TB pulmonary disease patients, was small. As contact investigation was performed for contacts of suspected pulmonary TB patients only, the number of index patients whose final diagnosis changed to another disease was small. Second, we could not perform subgroup analysis according to BCG vaccination status because of the small sample size, and therefore we could not confirm the effects of BCG vaccination on TST positivity. Third, we evaluated proximity according to 2 categories, that is, the same room versus a different room. Other factors, such as contact duration and intensity, could have affected the results. Finally, almost all patients underwent TST and IGRA in a single step. Therefore, it is possible that some contacts with negative results were in the window period during the time of contact investigation.

In conclusion, IGRA may be a better indicator of LTBI diagnosis than TST in household contacts of TB patients in countries where TB is prevalent and BCG vaccination mandatory. However, in elderly contacts, neither TST nor IGRA showed clear discrimination in positivity between TB and non-TB contacts. Further studies are needed to predict which elderly contacts are at risk for progression to active TB as well as to accurately detect recent *M tuberculosis* infection in this vulnerable population.

## Acknowledgments

The authors thank Biomedical Research Institute Grant (2016-2), Pusan National University Hospital for the support.
